# Inner Ear Morphology Is Perturbed in Two Novel Mouse Models of Recessive Deafness

**DOI:** 10.1371/journal.pone.0051284

**Published:** 2012-12-12

**Authors:** Kerry A. Miller, Louise H. Williams, Elizabeth Rose, Michael Kuiper, Hans-Henrik M. Dahl, Shehnaaz S. M. Manji

**Affiliations:** 1 Genetic Hearing Research, Murdoch Childrens Research Institute, Royal Children's Hospital, Melbourne, Victoria, Australia; 2 Department of Otolaryngology, University of Melbourne, Royal Victorian Eye and Ear Hospital, Melbourne, Victoria, Australia; 3 Victorian Life Sciences Computation Initiative, University of Melbourne, Melbourne, Victoria, Australia; 4 Department of Paediatrics, University of Melbourne, Melbourne, Victoria, Australia; 5 The HEARing CRC, Audiology, Hearing and Speech Sciences, University of Melbourne, Melbourne, Victoria, Australia; National Eye Institute, United States of America

## Abstract

Human *MYO7A* mutations can cause a variety of conditions involving the inner ear. These include dominant and recessive non-syndromic hearing loss and syndromic conditions such as Usher syndrome. Mouse models of deafness allow us to investigate functional pathways involved in normal and abnormal hearing processes. We present two novel mouse models with mutations in the *Myo7a* gene with distinct phenotypes. The mutation in *Myo7a^I487N/I487N^ ewaso* is located within the head motor domain of Myo7a. Mice exhibit a profound hearing loss and manifest behaviour associated with a vestibular defect. A mutation located in the linker region between the coiled-coil and the first MyTH4 domains of the protein is responsible in *Myo7a^F947I/F947I^ dumbo*. These mice show a less severe hearing loss than in *Myo7a^I487N/I487N^ ewaso*; their hearing loss threshold is elevated at 4 weeks old, and progressively worsens with age. These mice show no obvious signs of vestibular dysfunction, although scanning electron microscopy reveals a mild phenotype in vestibular stereocilia bundles. The *Myo7a^F947I/F947I^ dumbo* strain is therefore the first reported *Myo7a* mouse model without an overt vestibular phenotype; a possible model for human DFNB2 deafness. Understanding the molecular basis of these newly identified mutations will provide knowledge into the complex genetic pathways involved in the maintenance of hearing, and will provide insight into recessively inherited sensorineural hearing loss in humans.

## Introduction

A fully functional auditory system is required by humans to communicate and to perceive the surrounding environment. Disruption of this system, and the closely associated vestibular system, can lead to severe impairments to an individual's hearing and balance, and can be attributed to genetic and/or environmental factors. A highly heterogeneous trait, hearing loss is the most prevalent congenital sensory defect, where 1 in 500 newborns suffer from a considerable hearing impairment [1]. A moderate to severe hearing impairment can have a significant impact on speech, language and general development, incurring lifelong social, educational and economic costs [2]. Hearing loss can also be associated with additional clinical abnormalities, as seen in Pendred and Usher syndromes [3]–[5]. However in 70% of cases inherited hearing loss is non-syndromic, presenting as the only clinical feature [1], and in 80% of these cases is inherited in an autosomal recessive mode [6].

The mammalian ear is a highly complex and diverse organ. This is reflected in the extreme heterogeneity of inherited deafness. To date, 70 autosomal recessive loci have been mapped and 40 genes identified (http://hereditaryhearingloss.org), but there may be as many as 200 genes that contribute to this condition [7]. Several members of the myosin gene family have been implicated in hearing loss, including *MYO7A*, where mutations in humans are responsible for causing non-syndromic dominant (DFNA11; [8], [9]) and recessive (DFNB2; [10], [11]) deafness and the deaf-blindness condition Usher Syndrome type 1B (USH1B; [12]–[14]). Therefore it is evident that different mutations in *MYO7A* lead to differing phenotypic outcomes. The myosin motor superfamily of proteins consists of more than 20 distinct classes that regulate many cellular processes including the regulation of actin filament tension and cargo transportation [15], [16]. Myo7a is an unconventional myosin consisting of an N-terminal motor head domain that enables movement along actin filaments, and a neck and tail domain [1], [15]. It has a relatively restricted pattern of expression, detected in the testis, retina, lung, kidney and hair cells of the inner ear [17]–[19]. Mutations in this gene are reported to cause structural defects of the protein and consequently, auditory dysfunction [20].

Mouse models of disease provide insights into complex mammalian developmental and genetic pathways [21]. As the mammalian cochlea is highly conserved across species, mouse models are often used in the identification of genes involved in hearing loss and in the study of auditory processes and clinical features of genetic deafness [22]–[25]. The use of mutant mice generated using the alkylating agent *N*-ethyl-*N*-nitrosourea (ENU) has proven to be highly successful in the discovery and understanding of genes associated with human disease [26]–[28]. ENU randomly creates point mutations across the genome, meaning the observed phenotypes are likely to be a consequence of a single gene effect [29]. We undertook a comprehensive ENU mouse screen at the Australian Phenomics Facility (APF) to identify and characterise novel mouse models of recessively inherited hearing loss and present data on two novel mouse models of deafness with mutations in the *Myo7a* gene. Understanding the molecular basis of these individual mutations will provide insights into the complex genetic pathways involved in the development and maintenance of hearing.

## Materials and Methods

### Mice

Mice were screened for hearing loss in two independent screens from a large-scale ENU mutagenesis program at the APF, as described previously [25]. All mouse procedures were approved by the Royal Children's Hospital Animal Ethics Committee, RCH AEEC #A488 and #A585.

### Hearing Tests and Phenotypic Observations

Mice were screened for hearing loss initially using a clickbox and subsequently by Auditory Brainstem Response (ABR), as previously described [25]. Specific auditory stimulus in the form of broadband clicks was delivered in a range of decibel sound pressure levels (50–120 dB SPL). Data were analysed using a non-paired T-test and analysis of variance. Behaviour associated with vestibular dysfunction was determined by circling and head tossing/star-gazing observations. Six month old *Myo7a^I487N/I487N^ ewaso* and *Myo7a^F947I/F947I^ dumbo* mutants were filmed for 1 minute and movements tracked with a computerised image analyzer (Image Pro Plus 6.1; Media Cybernetics Inc).

### Mapping and Mutation Analysis

Affected *ewaso* mice were outcrossed to the CBA/H mapping strain and brother-sister progeny crossed to produce affected F2 offspring. Genomic DNA was isolated from tails of hearing and deaf littermates by Proteinase K digestion followed by phenol/chloroform extraction and used for homozygosity mapping and subsequent identification of candidate regions. DNA from 20 affected *ewaso* mice were analysed by genome wide scans using 120 microsatellite markers (AGRF, Australia), and mapping refined using an additional 45 mice with Amplifluor SNP arrays (APF). Deafness loci were mapped using methods described previously [25]. Using the UCSC genome browser [30] linkage intervals were examined for known or putative deafness genes and top candidate genes sequenced.

DNA from affected *dumbo* mice were screened for mutations in the known deafness genes *Tmc1* and *Myo7a*, by sequencing all exons, intron/exon boundaries and most of the 5′ and 3′ untranslated regions of these genes.

### PCR, Sequencing and Genotyping

Gene-specific primers were designed for amplification of all 49 exons of the *Myo7a* gene (ENSMUST00000107127) and DNA amplified with HotStar Taq polymerase (Qiagen) or GoTaq® Flexi DNA Polymerase (Promega) using standard PCR cycling conditions with an annealing temperature of 58°C. PCR products were sequenced with a BigDye™ v3.1 Terminator Cycle Sequencing Kit (Applied Biosystems) and products read using an ABI 3130xl capillary genetic analyser (Applied Biosystems). Sequencing chromatograms were compared to the published gDNA sequence using Mutation Surveyor (v2.60) software and any differences identified and determined for potential pathogenicity using Polyphen and SIFT [31], [32]. Conservation of Myo7a mutations were analysed using Clustal W [33]. *Myo7a* primer sequences are available on request.

Genomic DNA from all progeny of each strain was amplified as above. PCR primers were designed to disrupt or introduce a restriction enzyme site in the presence of the mutated nucleotide in each strain to produce a different pattern of DNA digestion for each genotype. Primer and enzyme information is detailed in Table S1.

### Molecular Modeling of *Myo7a* mutations

To determine whether the *ewaso* p.I487N mutation compromises the structure or function of the Myo7a protein the ATP-bound myosin II structure (pdb entry: 1W9J) was compared to the inactive myosin V structural residues 1 to 780 (pdb entry: 2DFS; http://www.rcsb.org/pdb/home/home.do) to model the structural impact. Models were solvated in 72×96×160A box of water containing ∼0.15 M NaCl and simulated for approximately 4 ns at 310K using NAMD molecular dynamics [34]. Molecular modeling images were generated using the VMD software package [35].

As the *dumbo* p.F947I residue is not contained within the 3D protein domain used for analysis of myosin II, and is outside the Myo7a tail crystal structure recently published [36], pathogenicity of the *dumbo* p.F947I mutation was estimated using Polyphen (http://genetics.bwh.harvard.edu/pph/) where structural query options were set to default. A PSIC score of >2.0 identifies that that particular mutation was never or almost never observed in that protein family and would be classified as ‘probably-damaging’, scores of 1.5 to 2.0 classified as ‘possibly damaging’, and scores of <1.5 as ‘benign’. A second algorithm, SIFT (http://sift.jcvi.org/) was also used to predict the effect of the *dumbo* p.F947I amino acid substitution on protein function. A SIFT BLink analysis was performed using Myo7a protein ID NP_032689.2.

### Tissue Collection

Mice were anaesthetised with isoflurane and culled by cervical dislocation according to the National Health and Medical Research Council Australian code of practice for the care and use of animals for scientific purposes (RCH AEEC approval #A488, #A585). Adult mouse cochleae and postnatal day 5 (P5) cochlear sensory epithelia were dissected and processed as described [24], [37], [38]. Vestibular sensory epithelia were dissected from the vestibule of P2–P5 mice by removal of the otolithic membrane and otoconia to expose the sensory epithelium of the saccule or utricle maculae.

Ossicles were dissected from half heads of adult (20–28 week old) *Myo7a^+/+^*, *Myo7a^I487N/I487N^ ewaso* and *Myo7a^F947I/F947I^ dumbo* mice. Briefly, the middle ear was exposed by dissection of the bulla and removal of the tympanic membrane, taking care not to damage the malleus underneath. The malleus, incus and stapes were removed from the middle ear with care, stored in PBS and photographed with a Leica DC200 camera (Leica Microsystems Ltd).

### Hematoxylin and Eosin (H&E) Staining

Cochleae were isolated from 4, 8 and 12 week wild-type, heterozygous and homozygous mice for each strain and processed for H&E staining as described previously [25]. A standard H&E protocol was followed with a 4–5 min incubation in hematoxylin and 45 sec staining in eosin, and mounted with Entellan® (Merck) or Pertex (HD Scientific). Images were taken on a Nikon Eclipse 80i microscope (Pathtech).

### Immunohistochemistry

P5 cochlear sensory epithelia were processed for immunohistochemistry as previously described [24] using a rabbit polyclonal anti-MyoVIIa primary antibody (1∶900; Abcam), an Alexa Fluor® 594-conjugated goat anti rabbit IgG secondary antibody (1∶2500; Molecular Probes) and Alexa Fluor® 488 phalloidin (1∶250; Molecular Probes). Rabbit IgG (Invitrogen) was used as an isotype control.

### Scanning Electron Microscopy (SEM)

Cochleae from P5, 2, 4 and 8 week old wild-type, heterozygous and homozygous mice from each strain were dissected, fixed and processed as previously described [24]. Vestibular sensory epithelia were dissected as above. Tissues were viewed using a Philips XL30 FE scanning electron microscope.

## Results

### Mice exhibit elevated hearing thresholds with or without vestibular dysfunction

**Figure 1:**
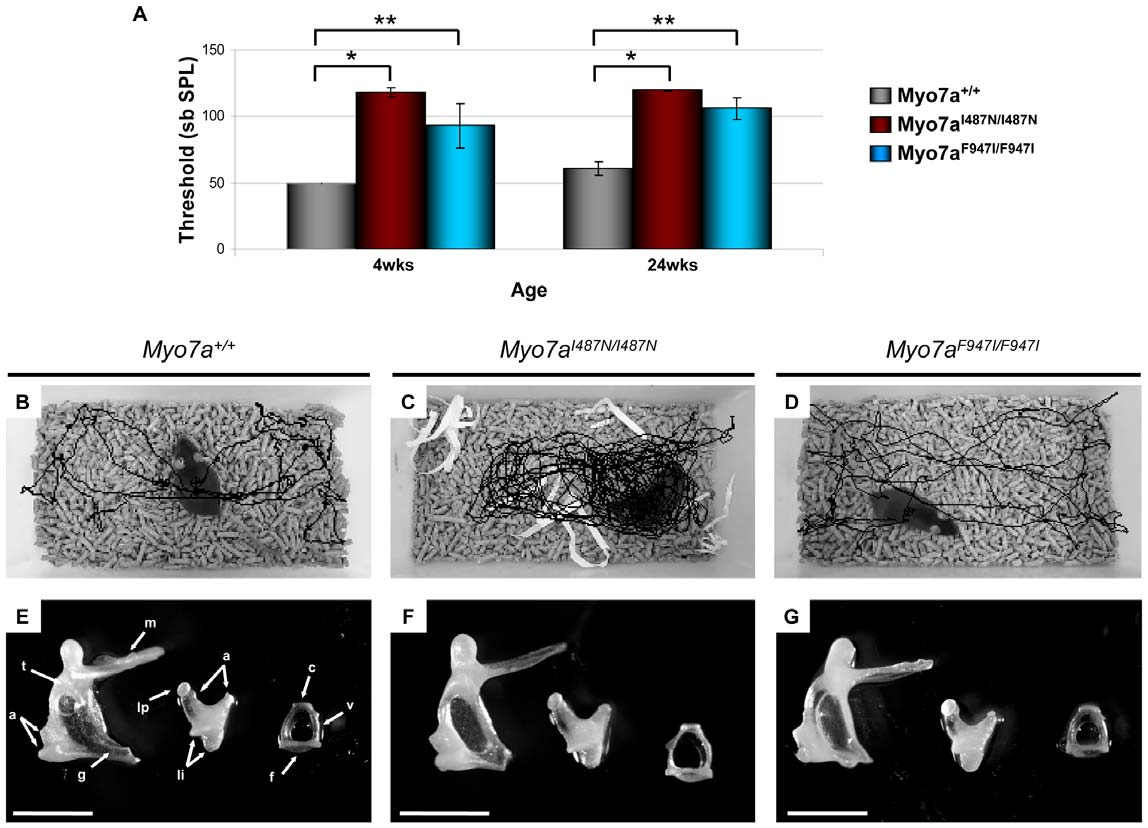
Phenotypic observations of *Myo7a* mutant strains. (**A**) Hearing profile of *Myo7a^+/+^*, *Myo7a^I487N/I487N^ ewaso* and *Myo7a^F947I/F947I^ dumbo* strains at 4 weeks (*p = 2.2×10^−25^, **p = 4.5×10^−10^) and 24 weeks (*p = 3.7×10^−29^, **p = 7.2×10^−20^). (**B–G**) Video surveillance and middle ear morphology in *Myo7a* strains. Observations highlighted an increased number of turns in *Myo7a^I487N/I487N^ ewaso* mice (**C**), when compared to wild-type (**B**). No such behaviour was seen in *Myo7a^F947I/F947I^ dumbo* mutants (**D**). Middle ear bones appear largely normal in *Myo7a^I487N/I487N^ ewaso* (**F**) and *Myo7a^F947I/F947I^ dumbo* (**G**) mutants, comparable to normal morphology of the malleus, incus and stapes (**E**). M; manubrium of malleus, A; articulation surfaces of malleus and incus joint, T; tubercle, G; gonial angle, LI; attachment points of suspensory ligaments of incus, LP; lenticular process, C; capitulum of stapes, V; arched ventral crus, F; footplate. Scale bar; 1 mm (E–G).

Homozygous *Myo7a^I487N/I487N^ ewaso* mice exhibit a profound hearing loss from 4 weeks of age (105–120 dB SPL; Figure 1A). Affected *Myo7a^I487N/I487N^ ewaso* mice were identified as having a vestibular dysfunction concomitant with their hearing loss, by exhibiting hyperactivity that manifested as circling/star-gazing behaviour (Figure 1C) and displayed abnormal trunk curling behaviour. An increase in the hearing threshold of *Myo7a^I487N/I487N^* mice at 24 wks can be attributed to inherent age related hearing loss in the C57BL/6 strain. However, *Myo7a^I487N/I487N^* heterozygous mice have a statistically significant increase in hearing threshold at 24 wks of age compared to wild-type littermates, indicating semi-dominance in this strain (Figure S1A).

In *Myo7a^F947I/F947I^ dumbo*, homozygote mice have a severe progressive hearing loss, with thresholds of 70–110 dB SPL at 4 weeks of age, reaching 95–110 dB SPL by 12 weeks (Figure 1A and S2), however these mice still retain some residual hearing at 24 weeks (90–110dB SPL; Figure 1A). Consistent with inherent age-related hearing loss in the C57BL/6 strain, wild-type and heterozygote mice also show an elevated hearing threshold by 24 weeks (Figure S1B). The behaviour in homozygous *Myo7a^F947I/F947I^ dumbo* mutants is consistent with a normal vestibular phenotype (Figure 1D).

Middle ear defects can also be associated with elevated ABR thresholds so to determine whether the hearing loss in *Myo7a^I487N/I487N^ ewaso* and *Myo7a^F947I/F947I^ dumbo* mutants was due to a disruption in conductance through the middle ear we examined these structures. In both *Myo7a^I487N/I487N^ ewaso* and *Myo7a^F947I/F947I^ dumbo* mutant mice there was no evidence of infection and the tympanic membrane and bulla were normal. Detailed examination of the ossicles did not highlight any structural differences of the malleus, incus or stapes in *Myo7a^I487N/I487N^ ewaso* (Figure 1F) and *Myo7a^F947I/F947I^ dumbo* (Figure 1G) mutants when compared to wild-type ossicles (Figure 1E). These findings all support a sensoreneural hearing loss in both *Myo7a^I487N/I487N^ ewaso* and *Myo7a^F947I/F947I^ dumbo* mutants.

### A *Myo7a* mutation is responsible for hearing loss in *ewaso* and *dumbo* mice

**Figure 2:**
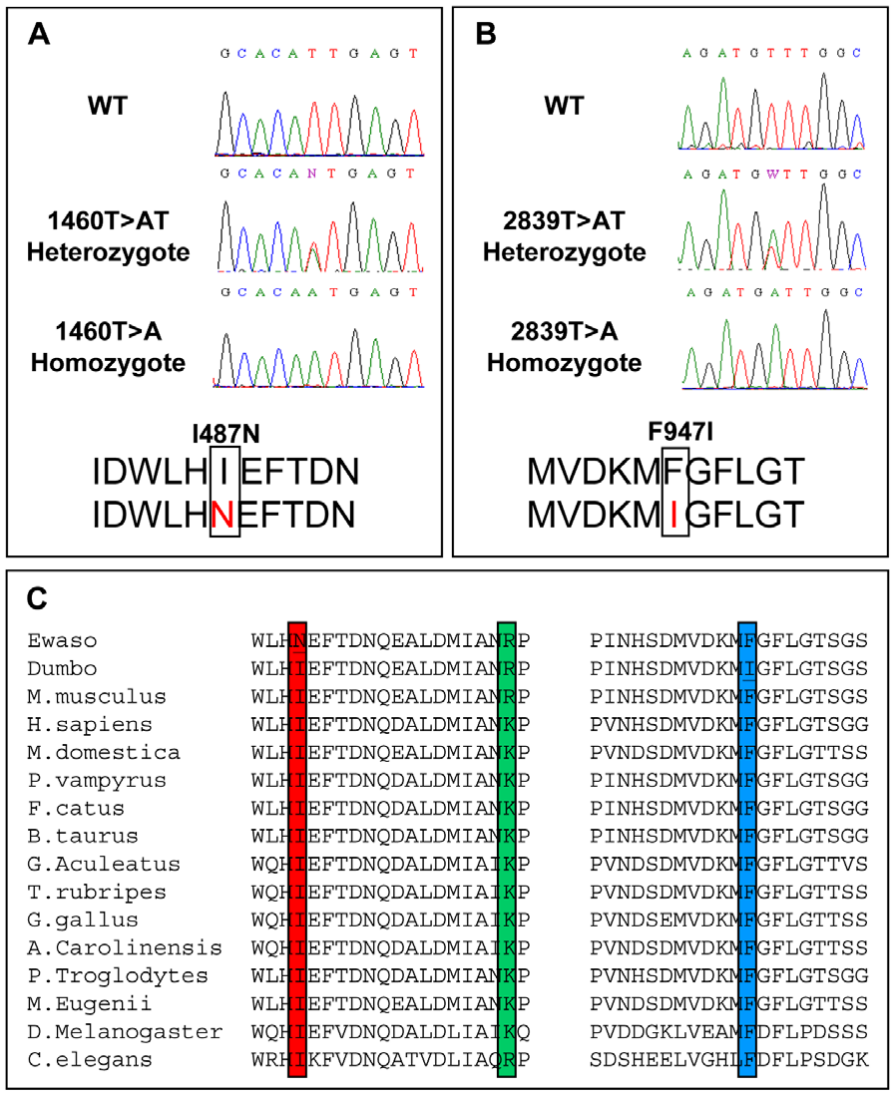
*Myo7a* mutations identified in *Myo7a^I487N/I487N^ ewaso* and *Myo7a^F947I/F947I^ dumbo* strains. (**A**) Direct sequencing identified a 1460T>A (I487N) missense mutation in *Myo7a^I487N/I487N^ ewaso* and a missense mutation 2839T>A (F947I) in the *Myo7a^F947I/F947I^ dumbo* mouse strain (**B**). Wildtype and heterozygote sequences are shown for comparison. (**C**) Sequence conservations of *Myo7a^I487N/I487N^ ewaso* (red) and *Myo7a^F947I/F947I^ dumbo* (blue) mutations, and the closely positioned *Myo7a^sh-1^ shaker* mutation (green) showing high evolutionary conservation of our two mutant strains.

The deafness locus in *Myo7a^I487N/I487N^ ewaso* was localised to a 7Mb region on mouse chromosome 7 by genome-wide homozygosity and fine mapping. The critical region was analyzed for known genes using multiple genome browsers (NCBI, http://www.ncbi.nlm.nih.gov; Ensembl, http://www.ensembl.org and UCSC, http://www.genome.ucsc.edu). This region contained the known deafness gene *Myo7a*. Direct sequencing of *Myo7a* revealed a novel T to A transversion at nucleotide position 1460 in exon 13, introducing an Ile to Asn substitution at position 487 in the protein (Figure 2A).

Sequencing of the *Myo7a* gene in *Myo7a^F947I/F947I^ dumbo* mice revealed a novel T to A change at nucleotide position 2839 in exon 23 that results in a Phe to Ile amino acid change at position 947 (Figure 2B).

Alignment of Myo7a protein sequences from 14 species identify that the amino acid residues affected by *Myo7a^I487N/I487N^ ewaso* and *Myo7a^F947I/F947I^ dumbo* mutations are highly conserved across evolution (Figure 2C). The causative mutation identified in these strains segregates with the deafness phenotype in all mice tested (n = 187/187 and 68/68, respectively).

### *In silico* Analysis indicates *Myo7a* mutations impair protein function

**Figure 3:**
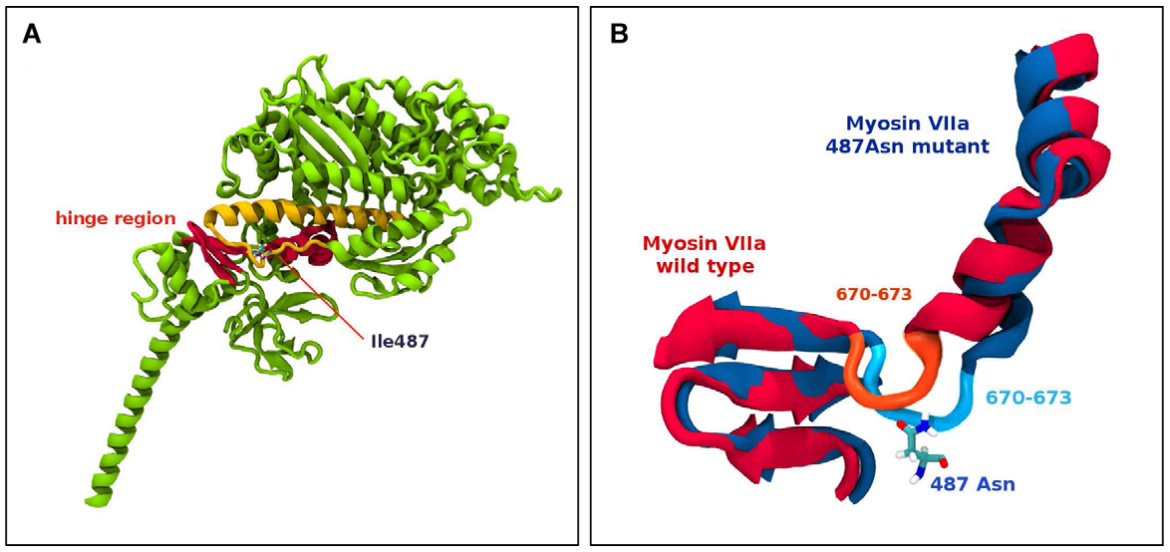
Molecular modelling of the *Myo7a^I487N/I487N^ ewaso* mutation. (**A**) Partial ribbon representation of a 3D model of myosin V 2DFS highlighting the Ile^487^ amino acid residue, which is localised to the hinge region between the head and tail domains of the protein. (**B**) Schematic diagram showing superimposed images of the hinge region (*Myo7a^+/+^* in red and the *Myo7a^I487N/I487N^ ewaso* mutation in blue) after 4 ns of molecular dynamics simulation showing a conformational change in the hinge region close to the Ile^487^ mutation site, showing the involvement of residues 670–673 around the hinge region causing the distortion.

The effect of the *ewaso* Myo7a p.I487N mutation on protein structure and function was evaluated using molecular modeling of wild-type and mutant Myo7a head domains, performed based on the 2DFS myosin V structure as a template of amino acid residues 1 to 780 (Figure 3A). A comparative dynamics study between the wild type and mutant form indicates a bulge region between residues 668 and 773 is destabilized by the presence of the Asn^487^ mutation. The mutant Asn^487^ residue is positioned to form hydrogen bonds with the protein backbone and also to Tyr^477^. Interaction of the mutant Asn^487^ with Tyr^477^ also appears to interfere with salt bridging interactions between Glu^473^, Arg^675^ and Arg^668^. Molecular modeling analysis predicts that the *ewaso* Myo7a p.I487N mutation induces a conformational change in the hinge region (amino acids 670–673) of the motor head domain that severely compromises the ‘power stroke” action of the protein.

Prediction output from PolyPhen for the *dumbo* Myo7a p.F947I mutation indicates a ‘probably damaging’ effect of the mutation on protein function, with a PSIC score of 2.001. SIFT prediction output denotes that both *ewaso* Myo7a p.I487N and *dumbo* Myo7a p.947I amino acid changes are ‘not tolerated’.

### Hair cell structure and stereocilia morphology are abnormal in *Myo7a* mutant strains

**Figure 4:**
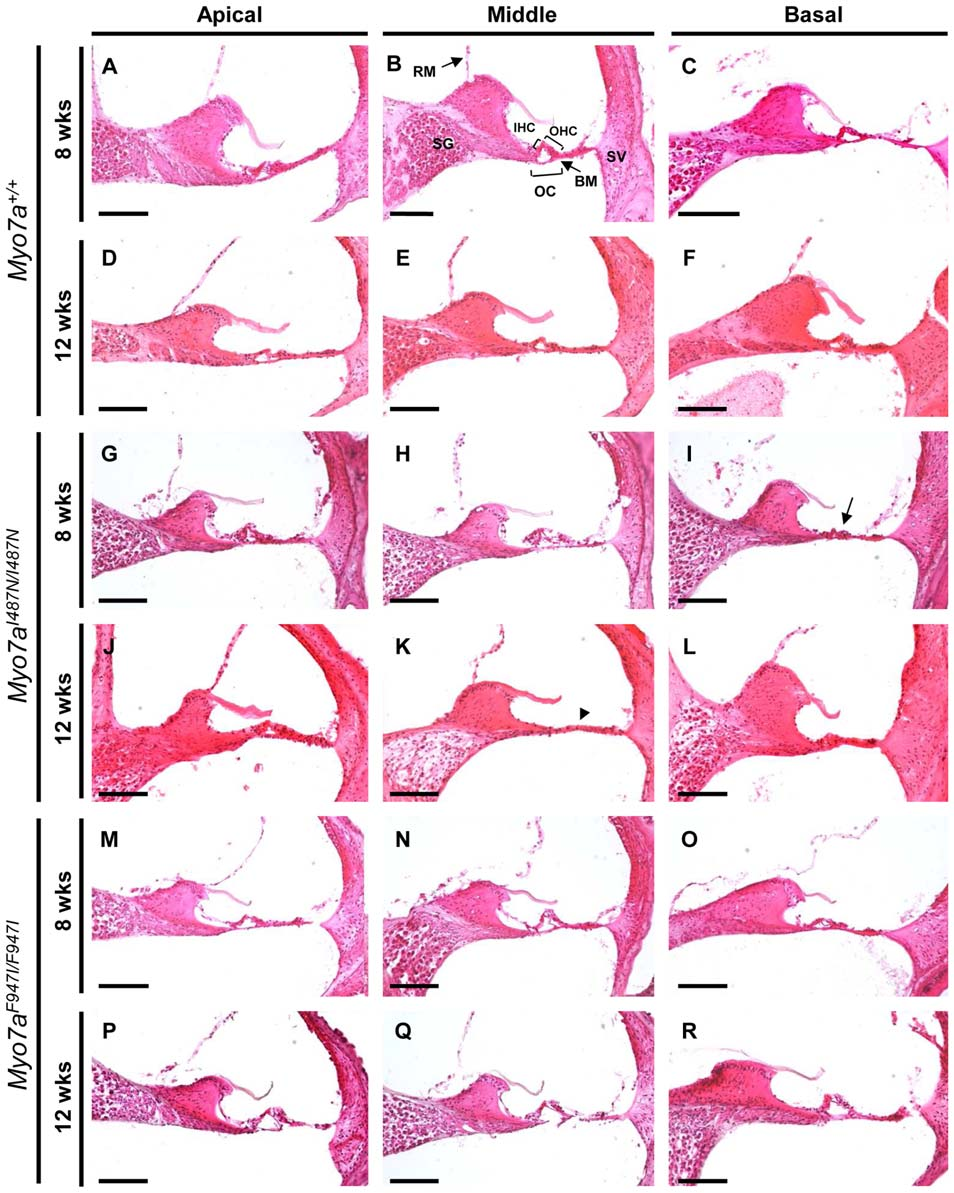
Haematoxylin and Eosin (H&E) staining of cochlear sections from *Myo7a* mutant strains at 8 and 12 weeks old. (**A–F**) *Myo7a^+/+^*, (**G–L**) *Myo7a^I487N/I487N^ ewaso* and (**M–R**) *Myo7a^F947I/F947I^ dumbo* mice at the apical, middle and basal levels. Normal cochlear morphology shows an intact organ of Corti and the presence of a tunnel containing inner and outer hair cells and intact spiral ganglion and stria vascularis (**B**). Early signs of cochlea degeneration are evident in the basal region of *Myo7a^I487N/I487N^ ewaso* mutant cochleae by 8 weeks of age (**I**), where the OC has collapsed (arrow in I). Complete lack of cellular architecture along the basilar membrane at the mid level is evident in this strain by 12 weeks (arrowhead in K). The cellular architecture in *Myo7a^F947I/F947I^ dumbo* mutants is largely normal at both 8 and 12 weeks. In both mutant strains the the spiral ganglion and the stria vascularis were normal at 8 and 12 weeks of age. RM, Reisner's membrane; SG, spiral ganglion; OHC, outer hair cells; IHC, inner hair cells; OC, organ of Corti; BM, basilar membrane; SV, stria vascularis. Scale bar; 100 µM.

No visible structural changes were identified on gross examination of the outer and middle ear structures (Figure 1B–G). Structural integrity of the inner ears was examined in H&E stained cochlear sections from 8 and 12 week old *Myo7a^I487N/I487N^ ewaso* and *Myo7a^F947I/F947I^ dumbo* revealing morphological differences in *Myo7a^I487N/I487N^ ewaso*, but not *Myo7a^F947I/F947I^ dumbo* mutants. Hair cell degeneration was observed in *Myo7a^I487N/I487N^ ewaso* mice at the basal cochlear region by 8 weeks of age (Figure 4I), evident by collapse of the organ of Corti. This becomes more pronounced by 12 weeks of age, where no cellular architecture is apparent at the mid cochlear level due to a lack of sensory or supporting cells in these mice (Figure 4K). In contrast, the sensory epithelium in *Myo7a^F947I/F947I^ dumbo* shows normal morphology (Figure 4M–R). No degeneration of the spiral ganglion or stria vascularis is evident in either strain.

**Figure 5:**
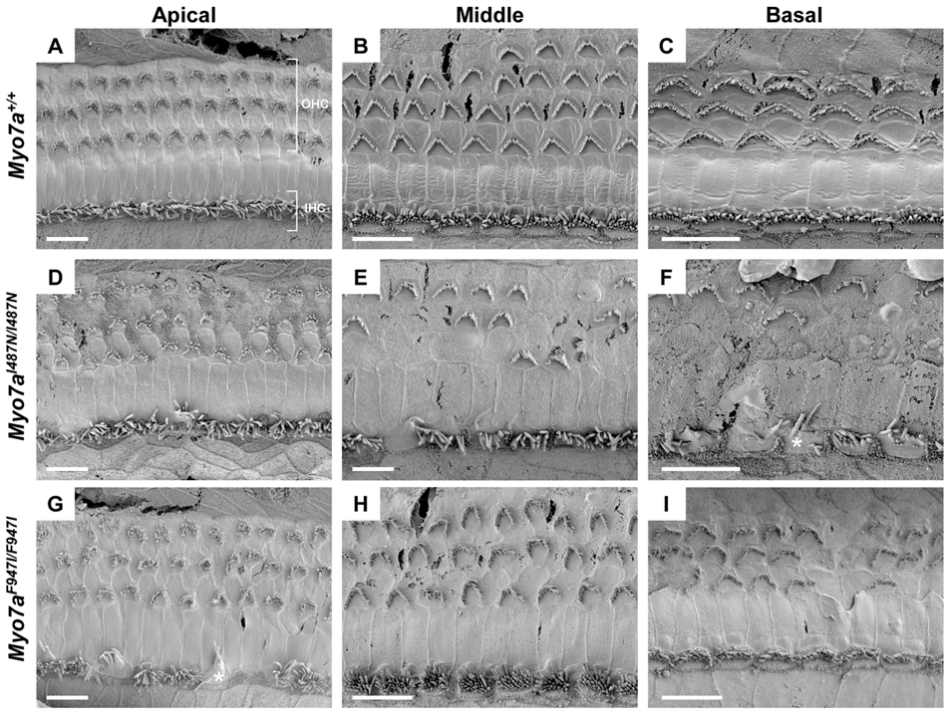
Scanning electron micrographs of cochlear sensory epithelium from *Myo7a* mutant strains at 8 weeks old. (**A–C**) *Myo7a^+/+^*, (**D–F**) *Myo7a^I487N/I487N^ ewaso* and (**G–I**) *Myo7a^F947I/F947I^ dumbo* mice at the apical, middle and basal cochlear level. Signs of degeneration and/or mis-orientation of OHC bundles is evident in both *Myo7a^I487N/I487N^ ewaso* (**D–F**) and *Myo7a^F947I/F947I^ dumbo* (**G–I**) mice at all levels of the cochlea. This appears to be more severe in *Myo7a^I487N/I487N^ ewaso* mutants, where many bundles are missing in the mid and basal regions (**E and F**). IHC bundles are also affected, appearing disorganised and/or showing signs of fusion in the basal levels of *Myo7a^I487N/I487N^ ewaso* cochleae (asterisk in F), and conversely in the apical region of *Myo7a^F947I/F947I^ dumbo* mutants (asterisk in G). Scale bar; 10 µM (A–I).

**Figure 6:**
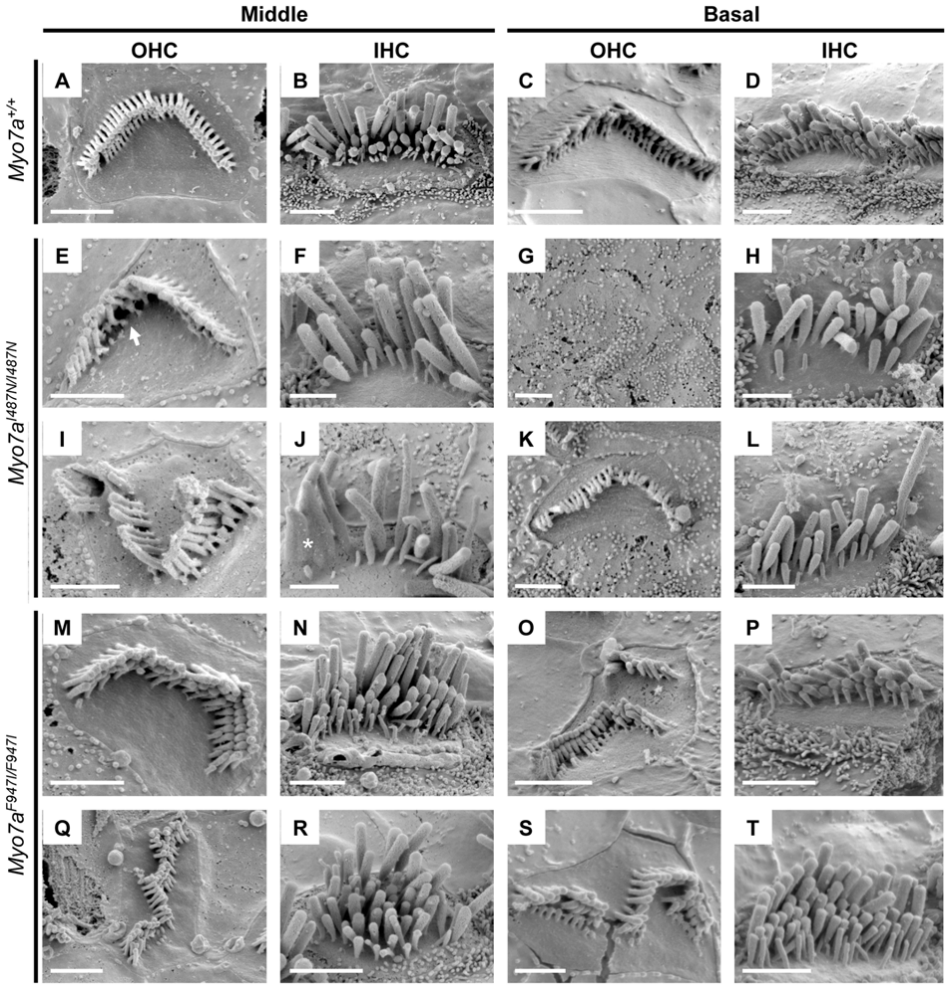
Scanning electron micrographs of inner (IHC) and outer (OHC) hair cells from 8 week old mice. (**A–D**) *Myo7a^+/+^*, (**E–L**) *Myo7a^I487N/I487N^ ewaso* and (**M–T**) *Myo7a^F947I/F947I^ dumbo* mice at the middle and basal cochlear level. OHC bundles of *Myo7a^I487N/I487N^ ewaso* mutants appear to have a regular V shaped array, however are missing the majority of their inner-row stereocilia at the middle level (arrow head in E). This is more severe at the basal level where many OHC bundles are missing (**G**). This phenotype is more severe in *Myo7a^F947I/F947I^ dumbo* mutants, where OHC bundles are disoriented, forming an array of OHC-type bundles (**M, Q, O and S**). Many *Myo7a^I487N/I487N^ ewaso* IHC bundles are affected at this age, with less numbers of stereocilia within the bundle and those remaining often showing signs of fusion (asterisk in J). The IHC bundles in *Myo7a^F947I/F947I^ dumbo* contain additional rows of stereocilia, however do still maintain a staircase-like structure (**N, R, P and T**). Scale bar; 2 µM.

Sensory organs of the cochlear and vestibular system were analysed by SEM. Examination of cochlear sensory epithelium in *Myo7a^I487N/I487N^ ewaso* showed mostly normal V-shaped outer hair cell (OHC) bundles at P5, although the occasional OHC was misorientated (Figure S3). Some inner hair cell (IHC) bundles showed abnormal morphology at the basal level at this age. At 2wks of age, IHC bundles at the mid and basal levels of the cochlea begin to show signs of disorganisation and/or fusion, and a few OHC bundles are misorientated, predominantly at the basal level (Figure S4). As this strain ages, the progression of abnormal stereocilia bundle morphology becomes evident. From 4 to 8 weeks increasingly more OHC bundles are affected, showing signs of degeneration of whole OHC bundles, as well as within bundles (Figures 5D–F, 6G, I and Figure S5). By 8 weeks large numbers of OHC bundles are missing at the basal and mid levels of the cochlea (Figure 5E and F). IHC bundles also show a progressive degeneration, with many appearing disorganised, comprising fused and often elongated stereocilia (Figure 5F and 6J).

In contrast to that seen in the *Myo7a^I487N/I487N^ ewaso* strain, *Myo7a^F947I/F947I^ dumbo* hair bundles appear to be more affected at the apical level of the cochlea. As early as P5, a number of OHC bundles are misorientated, however a few bundles also appear to be affected at the mid and basal levels (Figure S3). IHC begin to show abnormal structure by 2 weeks of age at the apical level and more OHC are misorientated at all levels of the cochlea (Figure S4). In addition to the progressive degeneration of IHC bundles, closer inspection of these bundles revealed the presence of additional rows of stereocilia (Figure 6N, R and T). OHC bundles become more severely misorientated with advancing age and some appear disintegrated in parts, forming individual ‘bundles’ (Figure 6O and S), At 8 weeks of age, the sensory epithelium of *Myo7a^F947I/F947I^ dumbo* mice shows minimal degeneration as a whole, as large numbers of OHC bundles are still present (Figure 5G–I).

**Figure 7:**
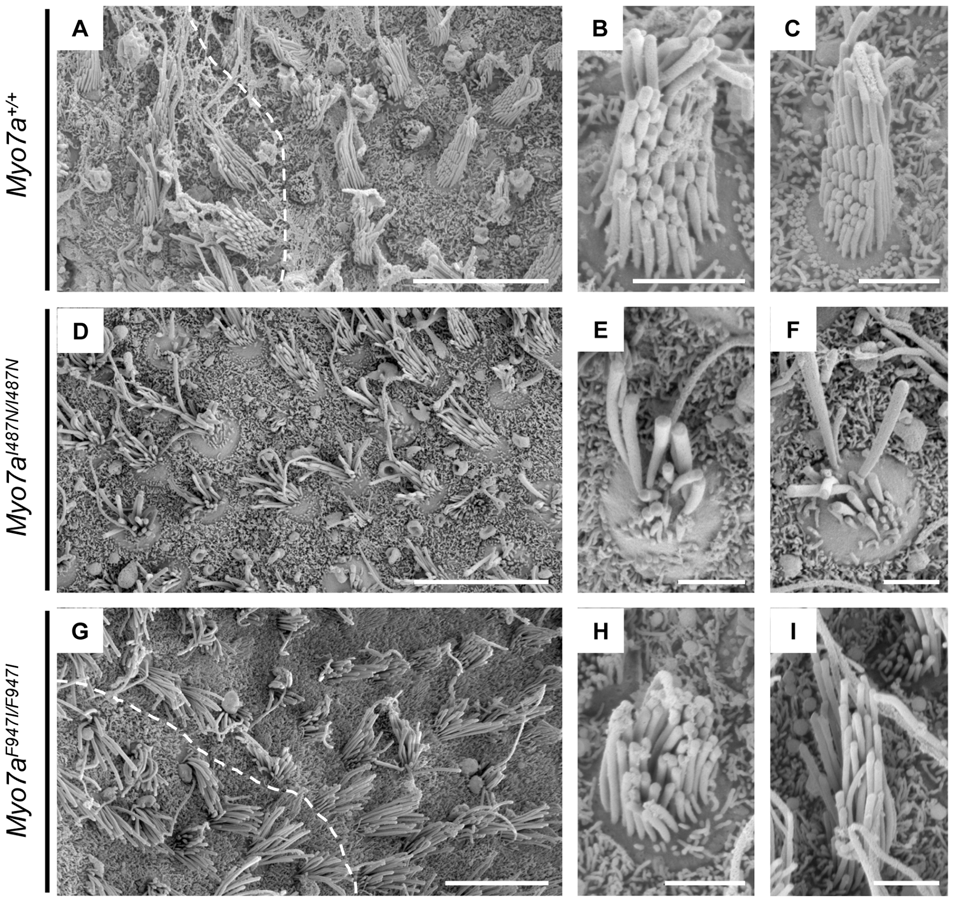
Scanning electron micrographs of P2–5 vestibular epithelia. (**A–C and J**) *Myo7a^+/+^*, (**D–F and K**) *Myo7a^I487N/I487N^ ewaso* and (**G–I and L**) *Myo7a^F947I/F947I^ dumbo*. A clear line of polarity reversal can be identified in *Myo7a^+/+^* saccular maculae (**A; dashed line**), and normal hair bundle morphology is apparent, with a staircase arrangement of stereocilia and kinocilium located with the tallest stereocilia (**B and C**). Hair cell bundles are affected in *Myo7a^I487N/I487N^ ewaso* saccules, and no clear zone of polarity is evident (**D**). These bundles do contain stereocilia of various lengths, however are generally disorientated and unstructured (**E and F**). A zone of polarity can be identified in *Myo7a^F947I/F947I^ dumbo* saccules (**G**), however hair bundles do appear to have a mild phenotype, with bundles missing some of the tallest (**H**) and/or shortest stereocilia (**I**). Scale bar; 10 µM (A, D and G), 2 µM (B, C, E, F, H and I).

Vestibular sensory epithelia were examined by SEM in *Myo7a^I487N/I487N^ ewaso* mice to determine the sensory organ morphology/defect underlying the circling/star-gazing behaviour in these mice. Normal saccular maculae consist of hair cells arranged in a particular orientation, such that on either side of the midline (striola) of the saccule, the hair cells (and stereocilia bundle) show opposite polarity (Figure 7A). These vestibular hair bundles contain several rows of stereocilia, arranged in a staircase orientation, with the longer stereocilia situated on the kinocilium side (Figure 7B and C). In *Myo7a^I487N/I487N^ ewaso* mutant mice, hair bundles are highly irregular, with rows of stereocilia missing and the staircase morphology disrupted (Figure 7E and F). The distorted hair bundles make identification of the zone of polarity reversal difficult in these mice (Figure 7D).

Vestibular sensory epithelia were also examined in *Myo7a^F947I/F947I^ dumbo* mutants, despite there being no overt vestibular phenotype evident in these mice. Saccular hair bundles in these mice appear largely normal, with an obvious staircase arrangement, however some of the tallest or shortest stereocilia are missing in some bundles (Figure 7H and I). The zone of polarity reversal however, is evident in *Myo7a^F947I/F947I^ dumbo* mutants (Figure 7G).

### Myo7a protein expression in *Myo7a* mutant strains

**Figure 8:**
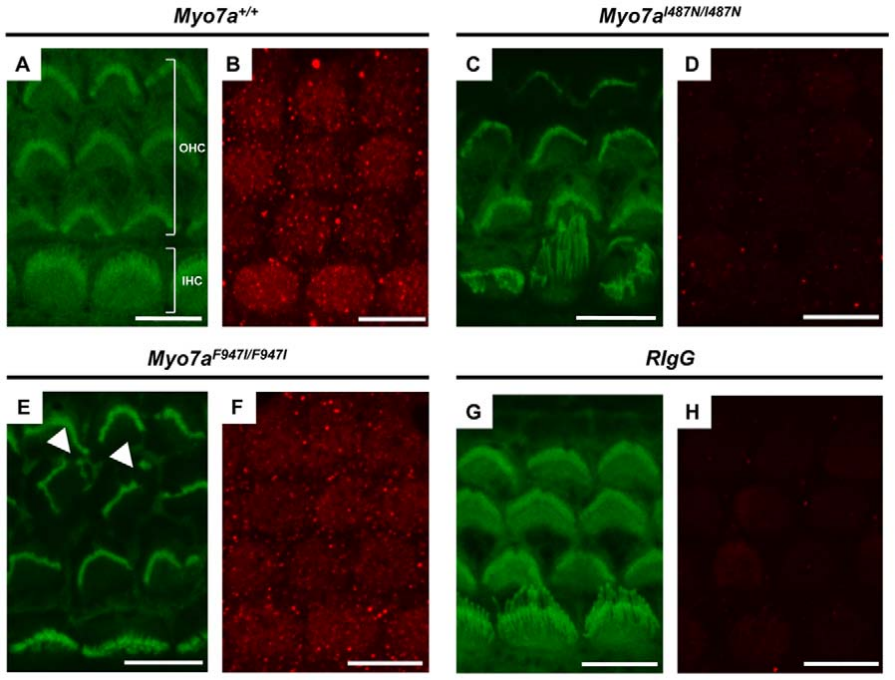
Immunohistochemistry of P5 sensory epithelia. Images of phalloidin (green) and Myo7a (red) stained cochlear sensory epithelium from P5 *Myo7a^+/+^* (**A and B**), *Myo7a^I487N/I487N^ ewaso* (**C and D**) and *Myo7a^F947I/F947I^ dumbo* (**E and F**) mice at the basal cochlear level. No Myo7a expression is evident in *Myo7a^I487N/I487N^ ewaso* mutant tissue (**D**), and may be slightly reduced in *Myo7a^F947I/F947I^ dumbo* mutants (**F**). No difference in protein localisation was observed between wilt-type and *Myo7a^F947I/F947I^ dumbo* tissue. Phalloidin staining highlights abnormal IHC structure in *Myo7a^I487N/I487N^ ewaso* mutants (**C**) and OHC hair bundles appear misorientated and/or fragmented in *Myo7a^F947I/F947I^ dumbo* (arrowheads in E). Scale bar; 8 µM (A–H).

The effect of *Myo7a^I487N/I487N^ ewaso* and *Myo7a^F947I/F947I^ dumbo* mutations on Myo7a protein expression was examined by immunofluorescence in P5 cochlear sensory epithelium. Myo7a protein localization was confirmed by confocal microscopy in the cytoplasm of inner and outer hair cells (Figure 8B). Reduced levels of Myo7a protein were observed in *Myo7a^F947I/F947I^ dumbo* sensory epithelium (Figure 8F), but levels of expression in *Myo7a^I487N/I487N^ ewaso* tissue appear to be undetectable, comparable to that seen in tissue incubated with an isotype control (Figure 8D and H).

## Discussion

It is well understood that mutations in the *MYO7A* gene can underlie certain forms of syndromic and non-syndromic deafness in the human population, specifically non-syndromic dominant (DFNA11) and recessive (DFNB2) deafness and Usher Syndrome type 1B (USH1B). Myosin VIIA was the first gene identified as a contributing factor to hearing loss in a genetic screen of mutations that lead to inner ear defects in the mouse [39], and until now 9 mouse models with mutations in the *Myo7a* gene have been published [19], [39]–[41]. We report two additional mouse models of deafness with novel mutations in the *Myo7a* gene, identified through an ENU mutagenesis screen. *Myo7a^I487N/I487N^ ewaso* mutant mice have a profound hearing loss with vestibular dysfunction due to a missense mutation affecting Ile residue 487, located in the motor head domain of the protein. The mutation identified in *Myo7a^F947I/F947I^ dumbo* results in a severe and progressive hearing loss but mice do not exhibit behaviour associated with a vestibular defect. This mutation lies in a linker region between the CC1 (coiled-coil) and first MyTH4 domains of the protein, the first reported in mice outside a functional/structural domain of the Myo7a protein.

**Figure 9:**
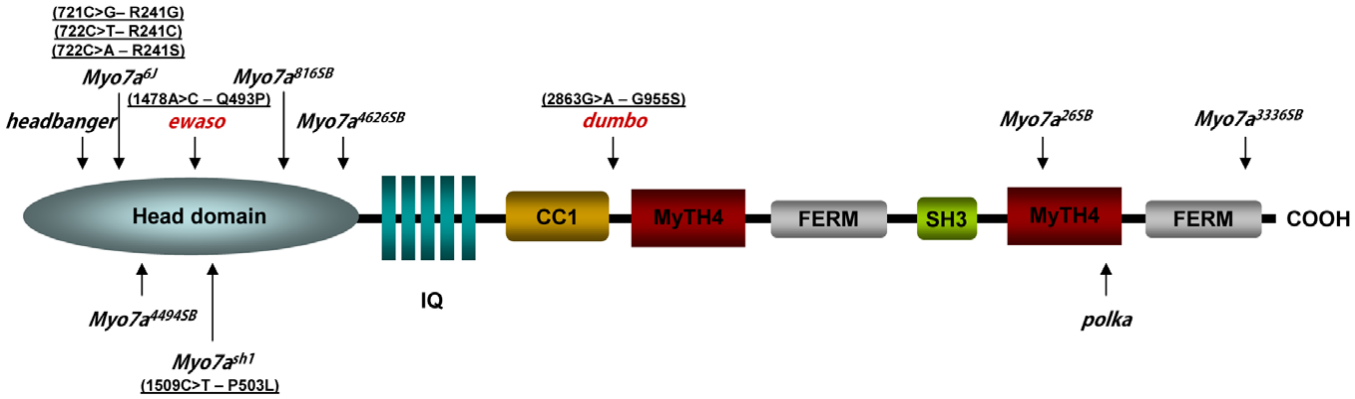
Schematic diagram of Myo7a protein structure showing the location of *Myo7a^I487N/I487N^ ewaso* and *Myo7a^F947I/F947I^ dumbo* mutations (in red) in relation to reported *shaker* mutations. DFNA11/DFNB2/USH1B human mutations within close proximity to a reported *shaker* mutation are shown in parentheses [14], [56], [57]. Details of these mouse mutations are included in Table 1. IQ, isoleucine-glutamine motif; CC1, Coiled Coil domain; MyTH4, Myosin Tail Homology 4; SH3, SRC Homology 3 domain.

The mouse Myo7a protein consists of a 729 aa N-terminal head domain, followed by a tail domain containing five light-chain binding IQ (isoleucine-glutamine) motifs, a predicted coiled-coil region (CC1) and two MyTH4-FERM repeats separated by an Src homology 3 domain (SH3; Figure 9, [42]). The head domain binds filamentous actin and undergoes a conformational change upon the hydrolysis of ATP, allowing it to “walk” along the actin structure and provide intracellular forces [43]. Previously reported *Myo7a* mouse models show a spectrum of phenotypes and mutations have been located across the protein (Figure 9, Table 1). The location and severity of these mutations correlate to the particular phenotype in each strain.

**Table 1:** Reported *Myo7a* mouse models.

*Mouse Model*	*Nucleotide change*	*Protein change*	*Exon*	*Phenotype*	*Affected domain*	*Reference*
***ewaso***	c.1460T>A	p.I487N	13	USB1B	head domain	This study
***dumbo***	c.2839T>A	p.F947I	23	DFNB2	linker region	This study
***headbanger***	c.531A>T	p.I178F	6	USB1B	head domain	[41]
***Myo7a^4494SB^***	c.592+2T>A	Truncation	In 6–7	USB1B	head domain	[40]
***Myo7a^6J^***	c.722G>C	p.R241P	7	USB1B	head domain	[39]
***Myo7a^sh1^***	c.1505G>C	p.R502P	13	USB1B	head domain	[39]
***Myo7a^816SB^***	c.1934-2A>G	Deletion of α-helix	In 16–17	USB1B	head domain	[39]
***Myo7a^4626SB^***	c.2158C>T	p.Q720X	18	USB1B	head domain	[40]
***Myo7a^26SB^***	c.5284T>A	p.F1762I	39	USB1B	MyTH4 2 domain	[40]
***polka***	c.5472+5G>A	Truncation	In 42–43	USB1B	MyTH4 2 domain	[19]
***Myo7a^3336SB^***	c.6432T>A	p.C2144X	48	USB1B	FERM domain	[40]

Our mouse models, *Myo7a^I487N/I487N^ ewaso* and *Myo7a^F947I/F947I^ dumbo*, show a variety of features identified in previously reported *Myo7a* strains. The phenotype observed in *Myo7a^I487N/I487N^ ewaso* reflects the severity of the highly conserved motor head domain mutation. Degeneration of OHC stereocilia in *Myo7a^I487N/I487N^ ewaso* at the mid to basal level of the cochlea is likely to be a consequence of the non-functional Myo7a protein. Molecular modeling data predicts that the *Myo7a^I487N/I487N^ ewaso* mutation affects the structure of the hinge region in the motor head domain in such a way that it severely compromises the ‘power stroke’ action of the protein. These mice also exhibit a severe vestibular phenotype supporting a major role of the motor head domain in Myo7a protein function. Hair cells of the vestibular sensory epithelium are severely disrupted, showing highly irregular staircase morphology and disruption of polarity, thereby disturbing normal linear and angular acceleration and affecting responses required for balance. This would indicate that stability and function of the mutant protein in the inner ear is severely affected in this strain.

*Myo7a^F947I/F947I^ dumbo* mutants have a less severe phenotype than seen in previously reported tail mutation strains *Myo7a^26SB^*, *Myo7a^3336SB^* and *polka* [19], [40]. These mice exhibit similarly disrupted morphology of the cochlear hair cells, although to a lesser degree. In the cochlea, the vibration of the basilar membrane in response to transmitted sound peaks at a location dependent on the sound frequency [42]. The apical to basal severity in *Myo7a^F947I/F947I^ dumbo* cochlea is most similar to that seen in *headbanger* homozygotes, and suggests residual hearing is maintained at higher frequencies (detected in the basal cochlear region) [41]. The mildly affected structure of vestibular hair bundles does not appear to influence balance in *Myo7a^F947I/F947I^ dumbo* mutants as no vestibular behaviour was observed using the methods described. This is the first such *Myo7a* mouse mutant not to exhibit such behaviour and may indicate the existence of an undetected or subtle vestibular phenotype in human patients with hearing loss that do not exhibit an obvious balance defect. It is also worth noting however, that some DFNB2 patients do exhibit some vestibular dysfunction [44]. The *Myo7a^F947I/F947I^ dumbo* mutation affects a highly conserved amino acid, and analysis by Polyphen and SIFT indicates a severe effect. This suggests that Myo7a may be compromised in its motor function due to disruption of the interactions that the Ile residue normally forms. Located several amino acids upstream of the MFS domain (MyTH4-FERM-SH3) it is likely to cause misfolding of the protein, therefore disrupting the Y-shaped architecture of this domain that in turn will disrupt the ability of Myo7a to interact with its scaffold protein sans [36], or in homodimer assembly [1]. It has been observed that in a sans mouse model of hearing loss stereocilia bundles also show a disrupted morphology, suggesting an interaction with Myo7a [45]–[47]. Data from *Myo7a^F947I/F947I^ dumbo* mice may also indicate that a direct interaction with an unknown protein found only in the auditory system is disrupted in these mice.

Myo7a is evidently required, either directly or indirectly, for maintaining the normal arrangement of stereocilia, and for hair bundle positioning at the top of the hair cell. In all *Myo7a* mouse models an abnormal array is seen across all levels of the cochlea and hair cell polarity defects are observed in many. In the inner ear, Myo7a is involved in transduction and adaption processes in the hair cells [48], controlling hair bundle organization, morphogenesis and polarity [49], [50], as well as in the elongation of stereocilia [51]. Our mouse models support this evidence as both *Myo7a^I487N/I487N^ ewaso* and *Myo7a^F947I/F947I^ dumbo* mutants exhibit defects that can be attributed to each of these processes. Protein function and/or interactions appear to be disrupted, thereby affecting signal transduction processes. This may be due to limitations in the physical positioning of hair bundles, or restricted bundle movement in response to external signals. Abnormal development of the hair cell bundle is possibly due to aberrant interactions with harmonin b and cadherin 23, required for development of a coherent structure [49]. Stereocilia reabsorption and abnormal array pattern is likely related to the detachment of individual stereocilia from the bundle via abnormal cross- or tip-links, where Myo7a is thought to play a role [48].

Human Usher syndrome is a dual sensory deficit disorder involving both the audiovestibular and visual systems [52] and in many cases a mutation in *MYO7A* will result in an Usher phenotype: congenital sensorineural hearing loss and retinitis pigmentosa identified by progressive loss of vision [52]. Many of these disease-causing mutations are located within the motor head domain of the protein, and the *Myo7a^I487N/I487N^ ewaso* mutant represents a model for this condition. Analysis of visual acuity and retinal histology in *Myo7a^I487N/I487N^ ewaso* and *Myo7a^F947I/F947I^ dumbo* mutant mice did not indicate any retinal phenotype (Miller et al, unpublished data). The lack of an eye phenotype is consistent with that seen in previously reported *Myo7a* mouse models, and several theories have been proposed to explain this observation such as alternative splicing and functional redundancy [53], [54]. However, a 9bp deletion in the coiled-coil domain in humans results in a moderate, progressive, non-syndromic hearing loss, a phenotype reflected in our *Myo7a^F947I/F947I^ dumbo* mutants that carry a mutation located just downstream of this domain [55].

The relationship between a particular mutation and its resulting phenotype is particularly important for improving our understanding of the molecular mechanisms involved in normal hearing, and is a pre-requisite for identifying possible therapeutic targets for sufferers of hearing loss. This requires the availability of mouse models with a range of mutations in a particular gene that recapitulate characteristics seen in humans. These two novel mouse models will facilitate the process of delineating the interactions, molecules and pathways involved in hearing loss. Our *Myo7a^F947I/F947I^ dumbo* strain is of particular interest, as it is the first characterised *Myo7a* mouse model without a vestibular dysfunction, and therefore the first for DFNB2.

## Supporting Information

Figure S1 Hearing profile of 4 and 24 wk (**A**) *Myo7a^+/+^* (n = 16, n = 13), *Myo7a^I487N/+^ ewaso* (n = 16, n = 22), *Myo7a^I487N/I487N^ ewaso* (n = 13, n = 18) and (**B**) *Myo7a^+/+^*, *Myo7a^F947I/+^ dumbo* (n = 13, n = 14) and *Myo7a^F947I/F947I^ dumbo* (n = 12, n = 25) mice at 24 weeks of age.*p = 9.8×10^−12^.
(TIF)

Figure S2 Hearing profile of *Myo7a^F947I/F947I^ dumbo* strain from 4 to 24 weeks of age. 4wk (n = 12), 8wk (n = 17), 12wk (n = 24), 24wk (n = 25). *p = 7.2×10^−20^.
(TIF)

Figure S3 SEM analysis of P5 cochlear sensory epithelium from Myo7a mutant strains. Apical, middle and basal cochlear turns were examined in Myo7a^+/+^ (A, B and C), Myo7a^I487N/I487N^ (D, E and F) and Myo7a^F947I/F947I^ (G, H and I) mice at P5. OHC, outer hair cells; IHC, inner hair cells. Stereocilia on the occasional OHC appear to be misorientated in *Myo7a^I487N/I487N^ ewaso* mice at this age (asterix in D), and more commonly in *Myo7a^F947I/F947I^ dumbo* mutants. Scale bar; 5 µM.
(TIF)

Figure S4 SEM analysis of 2 week old cochlear sensory epithelium from Myo7a mutant strains. Apical, middle and basal cochlear turns were examined in *Myo7a^+/+^* (A, B and C), *Myo7a^I487N/I487N^ ewaso* (D, E and F) and *Myo7a^F947I/F947I^ dumbo* (G, H and I) mice at 2 weeks. Misorientation of stereocilia bundles can be seen in both strains, particularly at the basal level in *Myo7a^I487N/I487N^ ewaso* mice (asterisk in F) and at all levels in *Myo7a^F947I/F947I^ dumbo* mutants. Fusion of stereocilia of IHC is also evident in *Myo7aI487N/I487N ewaso* at this age (arrowhead in F), and show abnormal structure in *Myo7a^F947I/F947I^ dumbo (asterisk in* G). OHC, outer hair cells; IHC, inner hair cells. Scale bar; 5 µM
(TIF)

Figure S5 SEM analysis of 4 week old cochlear sensory epithelium from Myo7a mutant strains. Apical, middle and basal cochlear turns were examined in Myo7a^+/+^ (A, B and C), Myo7a^I487N/I487N^ (D, E and F) and Myo7a^F947I/F947I^ (G, H and I) mice at 4 weeks. OHC, outer hair cells; IHC, inner hair cells. Scale bar; 10 µM.
(TIF)

Table S1 Primer and enzymes used in genotyping assays.
(DOC)
