# Supplementary figures and images for: Inner Ear Morphology Is Perturbed in Two Novel Mouse Models of Recessive Deafness
Source: PLoS One. 2012 Dec 12;7(12):e51284. doi: 10.1371/journal.pone.0051284 (PMC3520982; doi:10.1371/journal.pone.0051284)

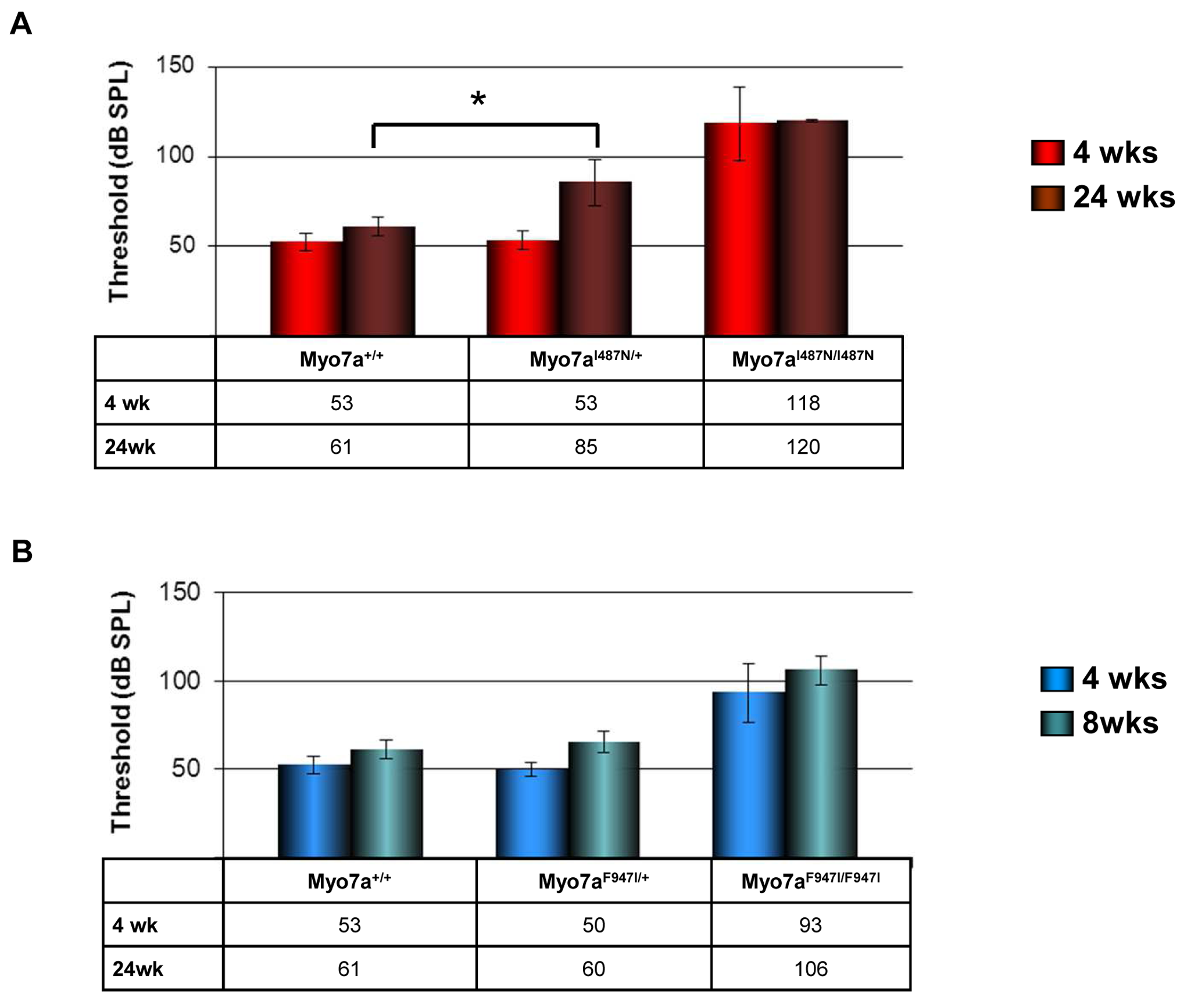

Supplement: Figure S1 — Hearing profile of 4 and 24 wk (A) Myo7a+/+ (n = 16, n = 13), Myo7aI487N/+ ewaso (n = 16, n = 22), Myo7aI487N/I487N ewaso (n = 13, n = 18) and (B) Myo7a+/+, Myo7aF947I/+ dumbo (n = 13, n = 14) and Myo7aF947I/F947I dumbo (n = 12, n = 25) mice at 24 weeks of age.*p = 9.8×10^−12^. (TIF) [file pone.0051284.s001.tif]

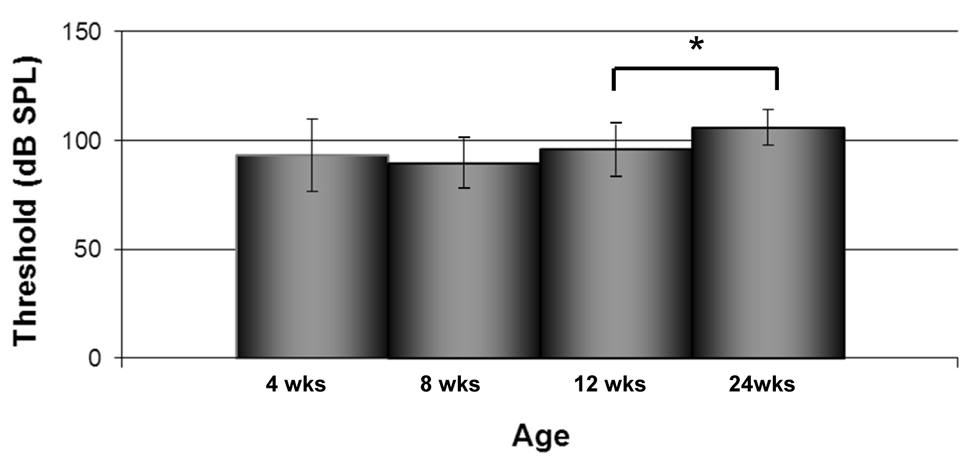

Supplement: Figure S2 — Hearing profile of Myo7aF947I/F947I dumbo strain from 4 to 24 weeks of age. 4wk (n = 12), 8wk (n = 17), 12wk (n = 24), 24wk (n = 25). *p = 7.2×10^−20^. (TIF) [file pone.0051284.s002.tif]

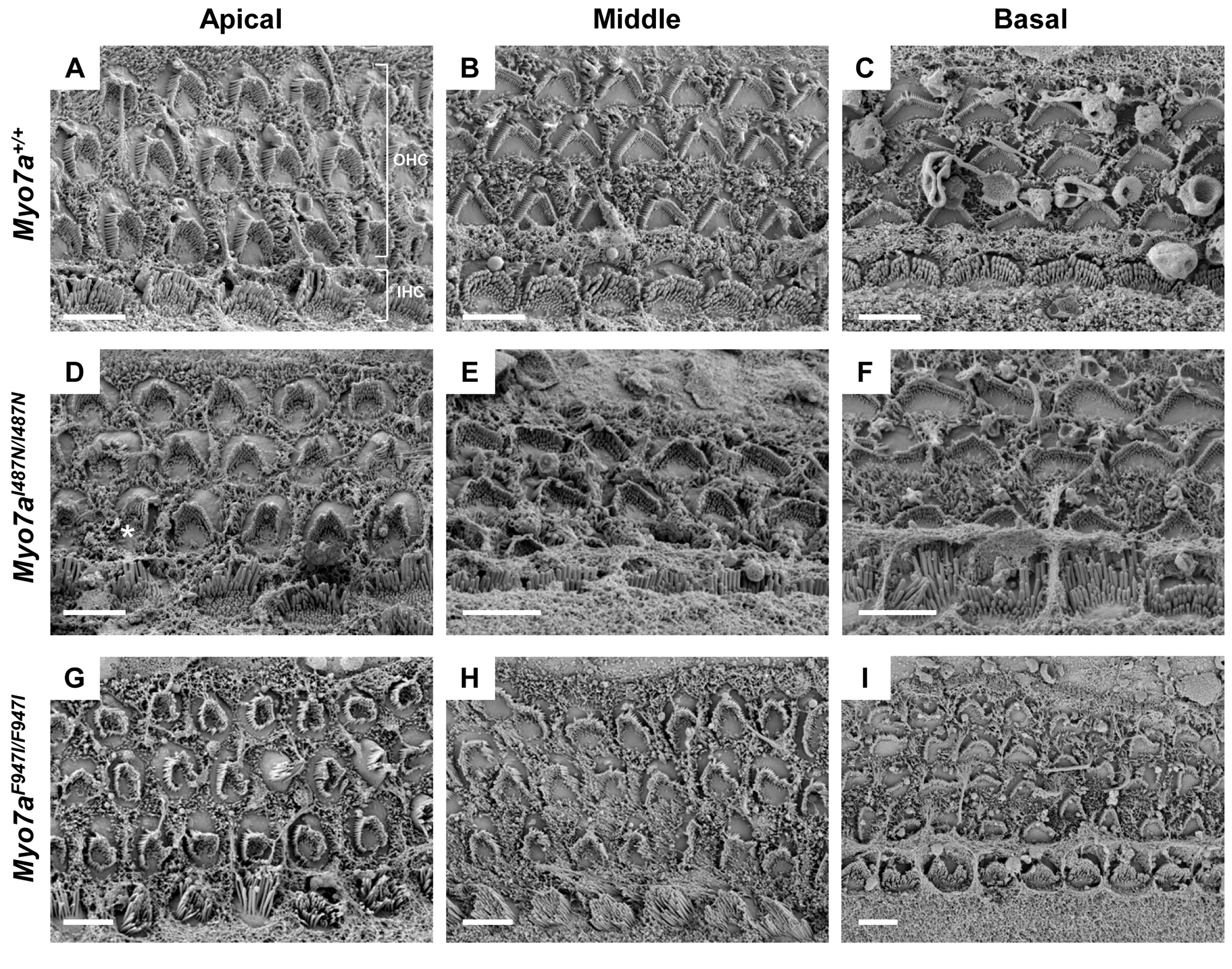

Supplement: Figure S3 — SEM analysis of P5 cochlear sensory epithelium from Myo7a mutant strains. Apical, middle and basal cochlear turns were examined in Myo7a+/+ (A, B and C), Myo7aI487N/I487N (D, E and F) and Myo7aF947I/F947I (G, H and I) mice at P5. OHC, outer hair cells; IHC, inner hair cells. Stereocilia on the occasional OHC appear to be misorientated in Myo7aI487N/I487N ewaso mice at this age (asterix in D), and more commonly in Myo7aF947I/F947I dumbo mutants. Scale bar; 5 µM. (TIF) [file pone.0051284.s003.tif]

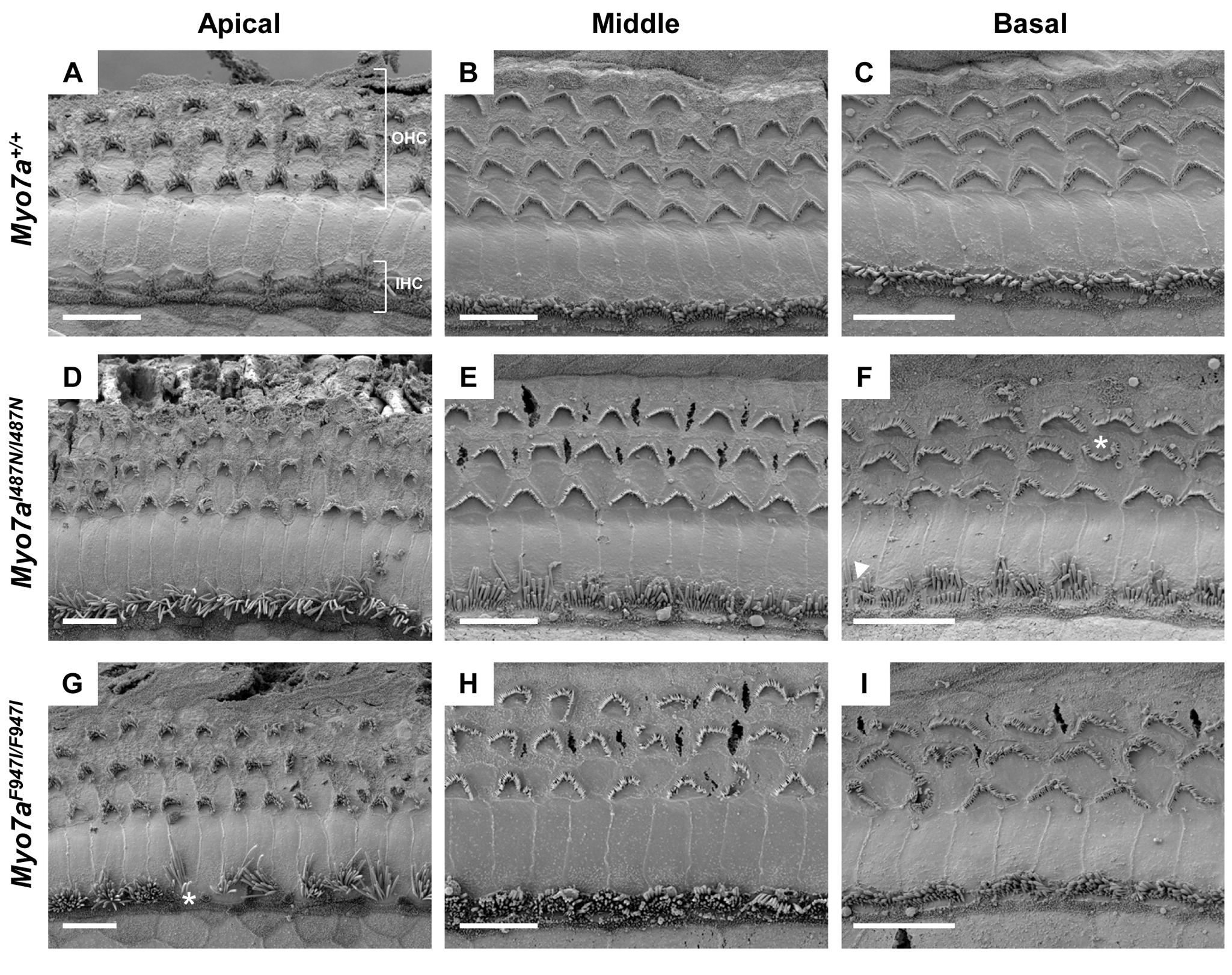

Supplement: Figure S4 — SEM analysis of 2 week old cochlear sensory epithelium from Myo7a mutant strains. Apical, middle and basal cochlear turns were examined in Myo7a+/+ (A, B and C), Myo7aI487N/I487N ewaso (D, E and F) and Myo7aF947I/F947I dumbo (G, H and I) mice at 2 weeks. Misorientation of stereocilia bundles can be seen in both strains, particularly at the basal level in Myo7aI487N/I487N ewaso mice (asterisk in F) and at all levels in Myo7aF947I/F947I dumbo mutants. Fusion of stereocilia of IHC is also evident in Myo7aI487N/I487N ewaso at this age (arrowhead in F), and show abnormal structure in Myo7aF947I/F947I dumbo (asterisk in G). OHC, outer hair cells; IHC, inner hair cells. Scale bar; 5 µM (TIF) [file pone.0051284.s004.tif]

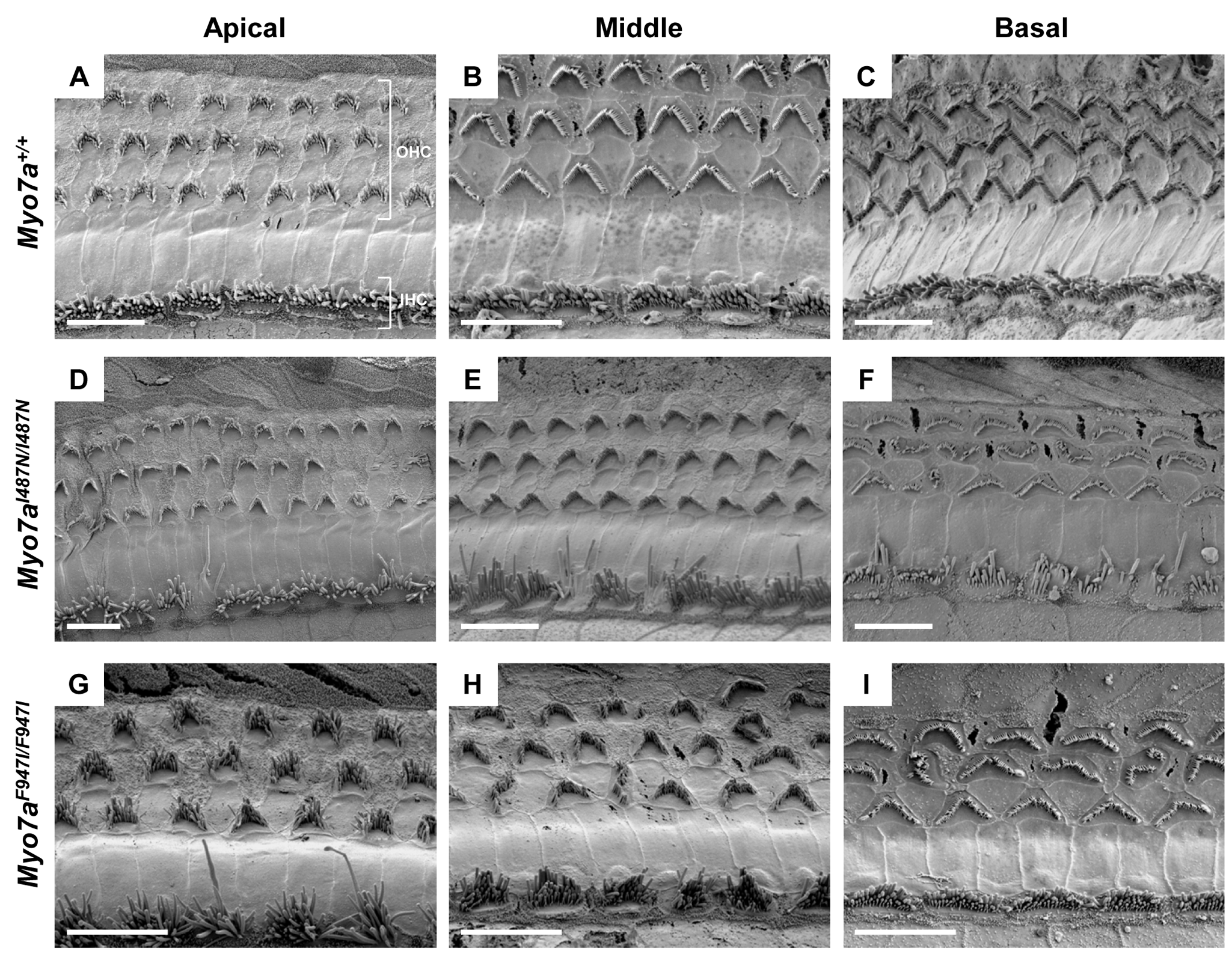

Supplement: Figure S5 — SEM analysis of 4 week old cochlear sensory epithelium from Myo7a mutant strains. Apical, middle and basal cochlear turns were examined in Myo7a+/+ (A, B and C), Myo7aI487N/I487N (D, E and F) and Myo7aF947I/F947I (G, H and I) mice at 4 weeks. OHC, outer hair cells; IHC, inner hair cells. Scale bar; 10 µM. (TIF) [file pone.0051284.s005.tif]
