# Supplementary material for: Inner Ear Morphology Is Perturbed in Two Novel Mouse Models of Recessive Deafness
Source: PLoS One. 2012 Dec 12;7(12):e51284. doi: 10.1371/journal.pone.0051284 (PMC3520982; doi:10.1371/journal.pone.0051284)
Supplement: Table S1 — Primer and enzymes used in genotyping assays. (DOC) [file pone.0051284.s006.doc]

**SUPPORTING INFORMATION**

**Table S1:** Primer and enzymes used in genotyping assays

| ***strain*** | ***Forward Primer*** | ***Reverse Primer*** | ***Enzyme*** |
| --- | --- | --- | --- |
| ***ewaso*** | 5’-CCACATGTCCAAGGTCCTCTTCC-3’ | 5’-CCAGTGCTTCCTGGTTGTCAGTGAACTCA-3’ | **MseI** |
| ***dumbo*** | 5’-CAACCACTCAGATATGGTGGACAAGATG-3’ | 5’-TGAGAATGGCTTCAGAGAGGGATAGC-3’ | **HpyCH4IV** |
